# The health and healthcare impact of providing insurance coverage to uninsured children: A prospective observational study

**DOI:** 10.1186/s12889-017-4363-z

**Published:** 2017-05-23

**Authors:** Glenn Flores, Hua Lin, Candice Walker, Michael Lee, Janet M. Currie, Rick Allgeyer, Alberto Portillo, Monica Henry, Marco Fierro, Kenneth Massey

**Affiliations:** 1 Medica Research Institute, MR-CW105, P.O. Box 9310, Minneapolis, MN 55440 USA; 2 Division of Health Policy and Management, University of Minnesota School of Public Health, 420 Delaware St SE, Minneapolis, MN 55455 USA; 3 Department of Pediatrics, University of Minnesota School of Medicine, 2450 Riverside Ave, Minneapolis, MN 55454 USA; 4 Department of Health Sciences Research, Mayo Clinic, 200 First Street SW, Rochester, MN 55905 USA; 5 Department of Clinical Sciences, University of Texas Southwestern Medical Center, 5323 Harry Hines Blvd, Dallas, TX 75390–9063 USA; 6 Texas Scottish Rite Hospital for Children, 2222 Welborn St, Dallas, TX 75219 USA; 7 Department of Pediatrics, University of Texas Southwestern Medical Center, 5323 Harry Hines Blvd, Dallas, TX 75390–9063 USA; 8 Woodrow Wilson School of Public and International Affairs, 316 Wallace Hall, Princeton University, Princeton, NJ 08544 USA; 9 Center for Strategic Decision Support, Texas Health and Human Services Commission, 4900 N. Lamar, Austin, TX 78751 USA; 10 University of Texas Southwestern Medical Center, 5323 Harry Hines Blvd, Dallas, TX 75390–9063 USA; 11 Department of Clinical Sciences, University of Texas Southwestern Medical Center, 5323 Harry Hines Blvd, Dallas, TX 75390–9063 USA

**Keywords:** Medically uninsured, Insurance, Medicaid, Children’s Health Insurance Program, Children, Adolescent, Hispanic Americans, African Americans, Poverty, Health policy

## Abstract

**Background:**

Of the 4.8 million uninsured children in America, 62–72% are eligible for but not enrolled in Medicaid or CHIP. Not enough is known, however, about the impact of health insurance on outcomes and costs for previously uninsured children, which has never been examined prospectively.

**Methods:**

This prospective observational study of uninsured Medicaid/CHIP-eligible minority children compared children obtaining coverage vs. those remaining uninsured. Subjects were recruited at 97 community sites, and 11 outcomes monitored monthly for 1 year.

**Results:**

In this sample of 237 children, those obtaining coverage were significantly (*P* < .05) less likely than the uninsured to have suboptimal health (27% vs. 46%); no PCP (7% vs. 40%); experienced never/sometimes getting immediate care from the PCP (7% vs. 40%); no usual source of preventive (1% vs. 20%) or sick (3% vs. 12%) care; and unmet medical (13% vs. 48%), preventive (6% vs. 50%), and dental (18% vs. 62%) care needs. The uninsured had higher out-of-pocket doctor-visit costs (mean = $70 vs. $29), and proportions of parents not recommending the child’s healthcare provider to friends (24% vs. 8%) and reporting the child’s health caused family financial problems (29% vs. 5%), and lower well-child-care-visit quality ratings. In bivariate analyses, older age, birth outside of the US, and lacking health insurance for >6 months at baseline were associated with remaining uninsured for the entire year. In multivariable analysis, children who had been uninsured for >6 months at baseline (odds ratio [OR], 3.8; 95% confidence interval [CI], 1.4–10.3) and African-American children (OR, 2.8; 95% CI, 1.1–7.3) had significantly higher odds of remaining uninsured for the entire year. Insurance saved $2886/insured child/year, with mean healthcare costs = $5155/uninsured vs. $2269/insured child (*P* = .04).

**Conclusions:**

Providing health insurance to Medicaid/CHIP-eligible uninsured children improves health, healthcare access and quality, and parental satisfaction; reduces unmet needs and out-of-pocket costs; and saves $2886/insured child/year. African-American children and those who have been uninsured for >6 months are at greatest risk for remaining uninsured. Extrapolation of the savings realized by insuring uninsured, Medicaid/CHIP-eligible children suggests that America potentially could save $8.7–$10.1 billion annually by providing health insurance to all Medicaid/CHIP-eligible uninsured children.

## Background

Approximately 44 million US children are covered by Medicaid or the Children’s Health Insurance Program (CHIP), which together insure half of low-income children in America [1, 2]. Forty-four percent of children with special healthcare needs have public insurance [1]. Medicaid covers 42% of US children (36 million), making it the single largest insurer of American children [2, 3]. CHIP covers 8.1 million children [3], 89% of whom reside in households with a family income ≤200% of the federal poverty threshold. Congress has extended federal CHIP funding three times since its enactment in 1997, most recently through September 2017 via the Medicare Access and CHIP Reauthorization Act of 2015 [4].

Of the 4.8 million uninsured children in America, 62–72% (3.0–3.5 million) are eligible for but not enrolled in Medicaid or CHIP [5–8]. Not enough is known, however, about the impact of Medicaid/CHIP coverage on outcomes for previously uninsured children, particularly regarding subsequent quality of care, parental satisfaction, out-of-pocket costs, family financial burden, and overall costs. Prior studies focused solely on CHIP’s impact on previously uninsured children demonstrated improved care access, health status, preventive-care utilization, and immunization rates [9–11]. But these studies were predominantly conducted in the 1990s/early 2000s, were pre/post telephone surveys without control groups, limited samples only to those who obtained insurance, and did not examine the impact of Medicaid. No prospective observational study (to our knowledge) has followed a cohort of Medicaid/CHIP-eligible uninsured children at baseline for 1 year to examine whether children who obtain coverage have better health and healthcare and lower costs than those remaining uninsured. Such a “natural history” study of outcomes for a cohort of uninsured children closely followed for 1 year would provide unique insights on the impact of Medicaid and CHIP on uninsured children and the risk factors for remaining uninsured.

The objective, therefore, was to conduct a prospective observational study of Medicaid/CHIP-eligible uninsured children to determine whether children obtaining coverage have better health, healthcare outcomes, and quality of care; greater parental satisfaction; lower family financial burden; and lower costs than children not obtaining coverage.

## Methods

### Research Design

This prospective observational study followed a cohort of uninsured, Medicaid/CHIP-eligible children for 1 year to assess the impact on children’s health and healthcare of obtaining CHIP, Medicaid, or other coverage vs. remaining uninsured. This study ran in parallel to a randomized, controlled trial (RCT) of an insurance intervention for uninsured children called Kids’ HELP©. Details on this RCT are reported elsewhere [12]. In the current prospective cohort study, an evaluation of a “natural experiment” was performed in which a cohort was assembled of minority children in low-income Dallas communities who had no health insurance but were verified to be eligible for Medicaid or CHIP. This cohort was then followed by the research team for 1 year to identify the impact of obtaining health insurance vs. remaining continuously uninsured on outcomes and costs. Thus, this prospective cohort design yields a “case” group of children who obtained insurance and a “control” group of children who remained uninsured after 1 year of follow-up. Study children potentially could obtain health insurance through two main mechanisms: voluntary, unassisted enrollment by the caregiver, or enrollment with the assistance of community-based organizations, the Kids’ HELP intervention (using Parent Mentors),^12^ or the staff at healthcare institutions (such as hospitals or clinics).

### Study Sites and Eligibility

Study sites consisted of five Dallas County communities with the highest proportion of uninsured and poor minority children [12]. Participant recruitment occurred at 97 community sites, including supermarkets, department stores, public libraries, Goodwill stores, food banks, health fairs, churches, schools, laundromats, and housing projects.

Eligibility criteria included that the child was: 1) of Latino or African-American race/ethnicity, by parental self-report; 2) 0–17 years old; 3) lacking health insurance at enrollment, but eligible for but not enrolled in Medicaid or CHIP; and 4) residing in Dallas County. Children’s Medicaid/CHIP eligibility was determined at enrollment by trained research staff using Texas eligibility criteria (including annual family income, number of children in the family, etc.) [13]. Both uninsurance status and Medicaid/CHIP eligibility were verified using the Texas Health and Human Services Commission’s (HHSC) electronic database.

### Measures

Insured children were defined as those who obtained health insurance at any time during the one-year follow-up period. Insurance coverage was verified using a three-step process: 1) initial parental report during monthly follow-up calls; 2) parents providing copies or photos of the insurance card or HHSC letter documenting coverage; and 3) confirmation of insurance and coverage date in the HHSC electronic database.

Outcomes included the child’s health status, health-related quality of life, access to healthcare, unmet medical and dental needs, use of health services, out-of-pocket costs of care (by monthly parental self-report), parental ratings of quality of the child’s healthcare, parental satisfaction with care, parental satisfaction with trying to obtain coverage (regardless of whether insurance ultimately was obtained), family financial burden, and missed school and work days due to children’s illness. Outcomes were evaluated using validated questions derived from previously published research and national, state, and regional surveys [9–11, 14–23].

### Data Collection

A trained, bilingual research assistant monitored outcomes via telephone calls or in-person visits using a standardized protocol, including 82-item baseline and 67-item 12-month follow-up questionnaires. Insurance coverage was monitored monthly for 12 months post-study-enrollment for all subjects. Other outcomes were evaluated at six and 12 months post-study-enrollment for all subjects, except parental satisfaction with trying to obtain coverage, assessed at 12 months post-study-enrollment. Participants received honoraria of $50 at enrollment, $5 for monthly follow-ups, and $10 for 6- and 12-month surveys.

### Analysis

SAS 9.1 was used for all analyses. Two research assistants conducted independent double data-entry. Bivariate analyses (nonparametric Wilcoxon and Pearson’s chi-square tests) evaluated the impact of health insurance on children’s health status, healthcare access, unmet healthcare needs, service use, care quality, parental satisfaction with care, out-of-pocket costs, family financial burden, and healthcare costs, as well as the association of baseline child and parent factors with the child remaining uninsured for the entire year. Two-tailed *P* values are reported, with *P* < .05 considered statistically significant. Stepwise multivariable logistic analyses were performed to examine factors associated with the child remaining uninsured for the entire year. The initial model included all variables (with an initial alpha-to-enter of 0.15); a second model was then constructed with only significant factors from the results of the first analysis; and the final model consisted of significant factors and relevant covariates, including primary caregiver employment, child born in the US vs. foreign born, and poverty.

A cost analysis was conducted to assess differences in societal costs for children obtaining health insurance vs. those remaining uninsured at one-year follow-up. Total costs consisted of direct medical and indirect costs associated with missed parental work days due to child illness. Direct medical costs were calculated by multiplying parental self-report of the number of children’s ED visits, hospital stays, and intensive-care-unit (ICU) stays by the mean medical costs of ED visits, hospital stays, and ICU stays at Children’s Health System of Texas. A recent study, using medical-record confirmation, demonstrated parental self-report is a valid and reliable method for assessing healthcare costs [24]. Costs for missed parental work days were calculated using employed caregiver’s hourly wages and other costs of caring for sick children (such as daycare-provider cost) multiplied by the number of missed work days.

## Results

### Baseline Sociodemographics

#### Characteristics of Children

Of 49,361 children screened, 49,032 were ineligible and excluded. Of the remaining 329, 63 subjects were excluded due to no longer being eligible for Medicaid or CHIP after initial screening, and 29 withdrew or were lost to follow-up (attrition rate = 8.8%), leaving a final sample of 237 participants who were confirmed eligible and successfully followed-up for 1 year. At baseline, the mean child age was 7 years, with equal gender distribution (Table 1). About two-thirds were Latino/Hispanic and one-third were African-American/black, most were US born, and approximately two-thirds had special healthcare needs. Ninety-five percent of children had prior coverage, predominantly Medicaid (75%), followed by CHIP (13%) and private insurance (11%), and children had been uninsured for a median of 6 months, ranging from 1 month to 9 years.

**Table 1:** Baseline sociodemographic characteristics of uninsured children and their parents (*N* = 237)

Characteristic	Mean or Proportion (*N* = 237)
Mean age of child, years (range)	7.3 (1–18)
Gender of child	
Male	119 (50%)
Female	118 (50%)
Race/ethnicity of child	
Latino/Hispanic	155 (65%)
African-American/black	82 (35%)
Child born in US	230 (97%)
Child has special healthcare needs	139 (59%)
Child ever had health insurance before	224 (95%)
Type of insurance uninsured child had in past	
Medicaid	169 (75%)
CHIP	30 (13%)
Private	24 (11%)
Instituto Mexicano del Seguro Social^a^	1 (0.5%)
Median no. of months child has been uninsured (IPR_95_^b^)	6 (1, 108)
Gender of primary caregiver	
Male	10 (4%)
Female	227 (96%)
Primary caregiver’s relationship to child	
Biological mother	215 (91%)
Biological father	8 (3%)
Other	14 (6%)
Race/ethnicity of primary caregiver	
Latino/Hispanic	151 (64%)
African-American/black	80 (34%)
White	6 (2%)
Primary caregiver’s educational attainment	
Not high-school graduate	150 (64%)
High-school graduate or at least some college	87 (36%)
Primary caregiver born in US	114 (48%)
Marital status of primary caregiver	
Married, living with spouse	86 (36%)
Single	49 (21%)
Married, separated from spouse	34 (14%)
Living with partner	22 (9%)
Common-law marriage	21 (9%)
Divorced	20 (8%)
Widowed	5 (2%)
Primary caregiver unemployed	130 (55%)
Primary caregiver has limited English proficiency	111 (47%)
Primary caregiver has health insurance	54 (26%)
Type of health insurance primary caregiver has	
Private	23 (43%)
Public	27 (52%)
Other	4 (5%)
Primary caregiver’s health status	
Excellent	32 (16%)
Very good	47 (23%)
Good	79 (39%)
Fair	38 (19%)
Poor	9 (4%)
Number of children in household	
1	67 (28%)
2	81 (34%)
≥ 3	89 (38%)
Number of adults in household	
1	71 (30%)
2	97 (41%)
≥ 3	69 (29%)
Mean combined annual family income, range	$22,060 ($0, $64,000)
Primary caregiver aware of child’s eligibility for Medicaid or CHIP	119 (52%)

^a^Public health insurance in Mexico

^b^IPR_95_ denotes inner 95th percentile range

#### Characteristics of Caregivers

Most caregivers were female and the biological mother, with races/ethnicities similar to their children (Table 1). Approximately two-thirds were not high-school graduates, half were US-born, and one-third were married and residing with the spouse. More than half were unemployed, and almost half had limited English proficiency. Only one-quarter had health insurance, with over half covered by Medicaid and 43% covered by private insurance. Over half had less than excellent/very good health status. About three-quarters of households had ≥2 children, and two-thirds had ≥2 adults. The mean combined annual family income was $22,060. Only 52% of caregivers were aware that their uninsured child was Medicaid/CHIP eligible.

### Baseline Health Status

At baseline, almost 40% of caregivers reported their children’s health status was not excellent/very good; more than three-quarters reported worrying about the child’s health more than other people and having emotional worry/concern about the child’s physical health, and the mean PedsQL score was 89.0 (Table 2).

**Table 2:** Health status, access to healthcare, unmet healthcare needs, use of services, quality of care, parental satisfaction with care, out-of-pocket costs, family financial burden, and costs of healthcare for uninsured children (*N* = 237) at baseline^a^

Outcome	Proportion or Mean (*N* = 237)
Health status	
Child’s health status not excellent or very good^b^	93 (39%)
Parent worries about child’s health more than other people	198 (84%)
Parent has emotional worry or concern about child’s physical health	182 (77%)
PedsQL total score	89.0 ± 13.2
Access to healthcare	
Child has no PCP	150 (63%)
Child has no usual source of preventive care	95 (40%)
Never/sometimes gets immediate care from PCP^c^	22 (21%)
Child has no usual source of sick care	41 (17%)
Different source of sick care and preventive care	127 (54%)
Has no 24-h telephone coverage for sick care	208 (88%)
Usual source of care has no night or weekend office hours	205 (87%)
Never/sometimes can obtain after-hours telephone help or advice^d^	12 (48%)
Unmet healthcare needs	
Delayed or didn’t get needed healthcare in past year	170 (72%)
Did not receive all needed preventive care^e^	88 (52%)
Did not receive all needed acute care^f^	105 (81%)
Did not receive all needed specialty care^g^	31 (58%)
Did not receive all needed dental care^h^	117 (61%)
Did not receive all needed prescription medications^i^	24 (18%)
Did not receive all needed medical supplies or equipment^j^	30 (49%)
Use of health services^k^	
Doctor visits in past year	3.3 ± 0.2
Preventive-care visits in past year	1.0 ± 0.1
Sick visits in past year	1.8 ± 0.2
ED visits in past year	0.9 ± 0.2
Hospital stays in past year	0.1 ± 0.1
Quality of pediatric care (scale of 1–10, where 10 = best)^l^	
Rating of overall quality of child’s well-child visit	8.3 ± 2.0
Rating of child’s PCP	8.9 ± 1.9
Rating of child’s acute care	8.6 ± 2.1
Rating of child’s specialty care	8.2 ± 3.5
Parental satisfaction with care	
Doctor never/sometimes takes time to understand child’s specific needs	52 (22%)
Doctor never/sometimes respects you are expert on your child	37 (16%)
Doctor never/sometimes asks how you are feeling as parent	97 (41%)
Doctor never/sometimes understands how you prefer to raise child	68 (29%)
Doctor did not spend enough time with child	36 (15%)
Did not ask all questions I wanted to ask	30 (15%)
Would not recommend child’s healthcare provider to friends	55 (23%)
Parental out-of-pocket costs of pediatric care^k^	
Out-of-pocket cost per doctor visit	$140 ± 35.7
Out-of-pocket cost per preventive-care visit	$46 ± 13.5
Out-of-pocket cost per sick visit	$195 ± 56.1
Out-of-pocket cost per ED visit	$434 ± 125
Out-of-pocket cost per hospital stay	$741 ± 476
Parental reported financial burden	
Need additional income to cover child’s medical expenses	102 (43%)
Child’s health caused financial problem for family	84 (36%)
Family cut down on work hours to obtain healthcare for child	51 (22%)
Caregiver stopped working because of child’s health	24 (10%)
Missed school and work days due to child’s health^k^	
Missed school days in past year	3.6 ± 0.4
Missed work days in past year due to child’s illness	1.6 ± 0.3
Wage loss in past year due to missed work days	$336 ± 77
Other costs in past year related to taking care of sick child	$167 ± 82

^a^Plus-minus values are means ±SD, except where noted. PCP denotes primary-care provider, and PedsQL^™^ denotes Pediatric Quality of Life Inventory Version 4.0 Generic Core Scales (scale of 0–100, where 100 is best score)

^b^By caregiver report

^c^Among the *N* = 104 who reported needing immediate care from the PCP

^d^Among the *N* = 25 who reported that they needed after-hours telephone help or advice

^e^Among the *N* = 170 who reported that their child needed preventive care

^f^Among the *N* = 129 who reported that their child needed acute care

^g^Among the *N* = 53 who reported that their child needed specialty care

^h^Among the *N* = 192 who reported that their child needed dental care

^i^Among the *N* = 132 who reported that their child needed prescription medications

^j^Among the *N* = 61 who reported that their child needed medical supplies or equipment

^k^Plus-minus values are means ±SE

^l^By caregiver report, using a scale of 0–10, in which 0 = worst possible rating and 10 = best possible rating

### Baseline Healthcare Access and Unmet Needs

Almost two-thirds of children had no PCP, 40% had no usual preventive-care source, and one in five never/sometimes obtains immediate PCP care (Table 2). Almost 20% of children had no usual source of sick care, over half had different sources of sick and preventive care, and 88% had no 24-h telephone coverage for sick care. Almost 90% reported the child’s usual source of care has no night/weekend office hours, and about half never/sometimes can obtain after-hours telephone help/advice regarding their child’s health. Almost three-quarters of parents delayed/didn’t get needed healthcare for their child in the past year, more than half didn’t receive all needed preventive care, and 81% had unmet acute-care needs. Over half of children had unmet specialty-care needs, about two-thirds had unmet dental-care needs, 18% didn’t receive all needed prescription medications, and half had unmet needs for medical supplies/equipment.

### Baseline Use of Health Services

At baseline, study children averaged three doctor visits, one preventive-care visit, two sick visits, one ED visit, and 0.1 hospital stays in the past year (Table 2).

### Baseline Quality of Care and Parental Satisfaction

Parent ratings of quality of pediatric care (1–10 scale, 10 = best) included mean scores of approximately eight for well-child visits and specialty care and nine for the PCP and acute care (Table 2). About one-quarter of caregivers reported the doctor never/sometimes takes time to understand the child’s specific needs and understands how you prefer to raise your child; 15–16% reported the doctor never/sometimes respects you as the expert on your child, didn’t spend enough time with the child, and I didn’t ask all questions I wanted to ask. Over 40% reported the doctor never/sometimes asks how you’re feeling as a parent, and about one-quarter wouldn’t recommend the child’s healthcare provider to friends.

### Baseline Financial Burden and Missed School/Work Days

Mean out-of-pocket costs were $140/doctor visit, $46/preventive-care visit, $195/sick visit, $434/ED visit, and $741/hospitalization (Table 2). Over one-third of caregivers reported needing additional income to cover children’s medical expenses and that the child’s health caused family financial problems, approximately one-quarter reported the family reduced work hours to obtain healthcare for the child, and one in 10 stopped working because of the child’s health. A mean of approximately four school days and two work days in the past year were missed due to children’s illness, resulting in a mean wage loss due to missed work days of $336, and mean other costs of $167 incurred caring for sick children.

### Outcomes at One-Year Follow-Up

#### Insurance Coverage and Health Status

At one-year follow-up, 196 children had obtained health insurance, most commonly Medicaid (76%) and CHIP (20%), with the remainder (4%) obtaining private coverage. Children remaining uninsured were almost twice as likely as those obtaining insurance to have not excellent/very good health status (46% vs. 27%, respectively; *P* = .01) (Table 3).

**Table 3:** Comparison of outcomes for uninsured vs. insured children at one-year follow-up^a^

Outcome	Proportion (%) or Mean for		*P*
	Uninsured Group (*n* = 41)	Insured Group (*n* = 196)	
Health status			
Health status not excellent/very good^b^	19 (46%)	53 (27%)	.01
Parent worries about child’s health more than other people	31 (76%)	133 (68%)	.35
Parent has emotional worry or concern about child’s physical health	27 (66%)	110 (56%)	.27
PedsQL total score	90 ± 11.4	89 ± 13.6	.59
Access to healthcare			
Child has no PCP	28 (68%)	36 (18%)	<.0001
Child has no usual source of preventive care	8 (20%)	1 (0.5%)	<.0001
Never/sometimes gets immediate care from PCP^c^	2 (40%)	3 (7%)	.02
Child has no usual source of sick care	5 (12%)	6 (3%)	.01
Different source of sick care and preventive care	23 (56%)	27 (14%)	<.0001
Has no 24-h telephone coverage for sick care	33 (80%)	111 (57%)	.004
Usual source of care has no night or weekend office hours	30 (73%)	119 (61%)	.13
Never/sometimes can obtain after-hours telephone help or advice^d^	1 (50%)	6 (29%)	.52
Unmet healthcare needs			
Delayed or didn’t get needed healthcare	20 (48%)	25 (13%)	<.0001
Didn’t receive all needed preventive care^e^	11 (50%)	9 (6%)	<.0001
Didn’t receive all needed acute care^f^	2 (25%)	4 (9%)	.17
Didn’t receive all needed specialty care^g^	1 (33%)	5 (18%)	.52
Didn’t receive all needed dental care^h^	16 (62%)	29 (18%)	<.0001
Didn’t receive all needed prescription medications^i^	0 (0%)	3 (3%)	.53
Didn’t receive all needed medical supplies or equipment^j^	6 (46%)	12 (23%)	.09
Use of health services^k^			
Doctor visits in past year	2.1 ± 0.5	2.9 ± 0.2	.01
Preventive-care visits in past year	0.7 ± 0.1	1.2 ± 0.1	.01
Sick visits in past year	1.2 ± 0.5	1.7 ± 0.2	.03
ED visits in past year	0.4 ± 0.2	0.3 ± 0.1	.91
Hospital stays in past year	0.05 ± 0.05	0.03 ± 0.02	.71
Quality of pediatric care (scale of 1–10, where 10 = best)^l^			
Rating of overall quality of child’s well-child visit	8.0 ± 0.3	8.9 ± 0.1	.01
Rating of child’s PCP	8.4 ± 0.5	9.2 ± 0.1	.16
Rating of child’s acute care	8.5 ± 0.4	9.1 ± 0.1	.17
Quality rating of child’s specialty care	6.8 ± 1.7	8.8 ± 0.3	.30
Parental satisfaction with care			
Doctor never/sometimes takes time to understand child’s specific needs	14 (34%)	28 (14%)	.01
Doctor never/sometimes respects you are expert on your child	12 (29%)	27 (14%)	.02
Doctor never/sometimes asks how you are feeling as parent	23 (56%)	91 (46%)	.26
Doctor never/sometimes understands how your prefer to raise child	17 (41%)	56 (29%)	.10
Doctor did not spend enough time with child	4 (10%)	19 (10%)	.99
Did not ask all questions I wanted to ask	5 (13%)	10 (5%)	.08
Would not recommend child’s healthcare provider to friends	10 (24%)	15 (8%)	<.0001
Satisfaction with process of obtaining insurance			<.0001
Very satisfied	4 (10%)	93 (48%)	
Satisfied	7 (18%)	71 (37%)	
Uncertain	11 (28%)	18 (9%)	
Dissatisfied	8 (20%)	7 (4%)	
Very dissatisfied	10 (25%)	5 (3%)	
Parental out-of-pocket costs of pediatric care^k^			
Mean out-of-pocket cost per doctor visit	$70 ± 15	$29 ± 15	<.0001
Mean out-of-pocket cost per preventive-care visit	$67 ± 29	$9 ± 3	.01
Mean out-of-pocket cost per sick visit	$86 ± 18	$16 ± 4	<.0001
Mean out-of-pocket cost per ED visit	$146 ± 75	$77 ± 63	.01
Mean out-of-pocket cost per hospital stay	$25 ± 25	$0 ± 0	.33
Parental-reported financial burden			
Need additional income to cover child’s medical expenses	12 (29%)	10 (5%)	.01
Child’s health caused financial problem for family	12 (29%)	13 (7%)	.01
Family cut down on work hours to obtain healthcare for child	3 (7%)	12 (6%)	.81
Stopped working because of child’s health	3 (7%)	5 (3%)	.12
Missed school and work days due to child’s health^k^			
Missed school days in past year	2.1 ± 0.4	2.5 ± 0.3	.34
Missed work days in past year due to child’s illness	0.5 ± 0.2	0.7 ± 0.2	.41
Wage loss in past year due to missed work days	$1237 ± 1038	$490 ± 229	.50
Other costs related to taking care of sick child	$6030 ± 5970	$622 ± 342	.02

^a^Plus-minus values are means ±SD, except where noted. PCP denotes primary-care provider, and PedsQL^™^ denotes Pediatric Quality of Life Inventory Version 4.0 Generic Core Scales

^b^By caregiver report

^c^Among the *N* = 48 who reported that their child needed immediate care from the PCP

^d^Among the *N* = 23 who reported that they needed after-hours telephone help or advice and had one-year follow-up data

^e^Among the *N* = 170 who reported that their child needed preventive care

^f^Among the *N* = 129 who reported that their child needed acute care

^g^Among the *N* = 53 who reported that their child needed specialty care

^h^Among the *N* = 192 who reported that their child needed dental care

^i^Among the *N* = 132 who reported that their child needed prescription medications

^j^Among the *N* = 61 who reported that their child needed medical supplies or equipment

^k^Plus-minus values are means ±SE

^l^By caregiver report, using a scale of 0–10, in which 0 = worst possible rating and 10 = best possible rating

#### Healthcare Access and Unmet Needs

Uninsured children were almost four times more likely than children obtaining insurance to have no PCP (68% vs. 18%; *P* < .01), 40 times more likely to have no usual preventive-care source (20% vs. 0.5%; *P* < .01), and significantly more likely to never/sometimes get immediate PCP care, have no usual source of sick care, have different sources of sick and preventive care, and have no 24-h telephone coverage for sick care (Table 3). Uninsured children were over three times more likely than the insured to delay/not get needed healthcare (48% vs. 13%; *P* < .01), eight times more likely to have unmet preventive-care needs (50% vs. 6%; *P* < .01), and had triple the likelihood of unmet dental-care needs (62% vs. 18%; *P* < .01). No other significant intergroup differences were noted in health status, access, or unmet needs.

#### Use of Health Services

Compared with children obtaining insurance, uninsured children averaged significantly fewer doctor visits, preventive-care visits, and sick visits in the past year (Table 3).

#### Quality of Care and Parental Satisfaction

The quality of well-child visits was rated lower by parents of uninsured vs. insured children (mean rating ± SD = 8.0 ± 0.3 vs. 8.9 ± 0.1) (Table 3). In several domains, parents of uninsured children had significantly greater dissatisfaction with their child’s care than parents of insured children, including the doctor never/sometimes takes time to understand the child’s specific needs (34% vs. 14%; *P* = .01), the doctor never/sometimes respects you’re the expert on your child (29% vs. 14%; *P* = .02), and the parent wouldn’t recommend the child’s healthcare provider to friends (24% vs. 8%; *P* < .01). Parents of uninsured children also were more than six times more likely to be dissatisfied or very dissatisfied with the process of trying to obtain health insurance for their children, at 45% vs. 7% (*P* < .0001). No other significant intergroup differences were found for service use, quality, or satisfaction.

#### Financial Burden and Missed School/Work Days

Mean out-of-pocket costs of care were significantly higher for parents of uninsured children vs. parents of children obtaining insurance, including for doctor visits (mean ± SE = $70 ± 15 vs. $29 ± 15, respectively; *P* < .0001), preventive-care visits ($67 ± 29 vs. $9 ± 3, respectively; *P* = .01), sick visits ($86 ± 18 vs. $16 ± 4, respectively; *P* < .0001), and ED visits ($146 ± 75 vs. $77 ± 63, respectively; *P* = .01) (Table 3). Compared with parents of insured children, parents of uninsured children were almost six times more likely to report needing additional income to cover their children’s medical expenses (29% vs. 5% among parents of the uninsured; *P* = .01) and more than four times more likely to report that the child’s health caused financial problems for the family (29% vs. 7%; *P* = .01). The mean (±SD) annual costs of caring for sick children also were significantly higher for parents of uninsured children vs. those with insured children ($6030 ± 5970 vs. $622 ± 342; *P* = .02). No other significant intergroup differences were noted for financial burden or missed school/work days.

#### Factors Associated with Remaining Uninsured for the Entire Year

In bivariate analyses, older child age, being foreign-born, and being uninsured for a greater number of months at baseline were associated with remaining uninsured for the entire year (Table 4). In multivariable analyses (Table 5), being uninsured for >6 months at baseline was associated with approximately four times the adjusted odds (odds ratio [OR], 3.8; 95% confidence interval [CI], 1.4–10.3) and African-American race/ethnicity approximately triple the adjusted odds (OR, 2.8; 95% CI, 1.1–7.3) of remaining uninsured for the entire year. Child age was no longer a significant predictor of persistent uninsurance after adjustment for relevant covariates, and therefore did not enter the final model. The child being foreign-born and child gender were no longer significant predictors of persistent uninsurance after adjustment for caregiver employment and combined family income.

**Table 4:** Comparison of baseline child and parental characteristics of children who remained uninsured vs. those obtaining health insurance at one-year follow-up (*N* = 237)

Characteristic	Mean or % At Baseline		
	Uninsured (*N* = 41)	Insured (*N* = 196)	*P*
Mean age of child, years (range)	9.4 (1–18)	6.9 (1–18)	<.01
Child born in US	90%	98%	<.01
Mean months without insurance (range)	21.2 (2–108)	12.7 (1–132)	.02
Median months without insurance (IPR_95_^a^)	15 (1, 108)	6 (1, 108)	.02
Child has special-care needs	71%	56%	.08
Gender of child			.12
Male	61%	47%	
Female	39%	53%	
Type of insurance that uninsured child had in past			.12
Medicaid	73%	76%	
CHIP	16%	12%	
Private	8%	12%	
Instituto Mexicano del Seguro Social^b^	3%	0%	
Race/ethnicity of primary caregiver			.13
Latino	66%	64%	
African-American	32%	34%	
White	2%	2%	
Primary caregiver’s relationship to child			.17
Mother	85%	92%	
Father	2%	4%	
Other	12%	5%	
Child ever had health insurance before	90%	95%	.19
Primary caregiver has health insurance	35%	25%	.20
Mean combined annual family income, (range)	$24,078 ($42,00, $56,000)	$21,648 ($0, $64,000)	.24
Gender of primary caregiver			.28
Male	7%	4%	
Female	93%	96%	
Primary caregiver has limited English proficiency	54%	45%	.33
Primary caregiver born in US	41%	49%	.35
Marital status of primary caregiver			.46
Married, living with spouse	46%	34%	
Single	15%	22%	
Married, separated from spouse	17%	14%	
Living with partner	2%	10%	
Common-law marriage	7%	9%	
Divorced	7%	9%	
Widowed	6%	2%	
Primary caregiver’s health status			.55
Excellent	10%	17%	
Very good	16%	24%	
Good	42%	38%	
Fair	26%	17%	
Poor	6%	4%	
Primary caregiver aware that child is eligible for Medicaid or CHIP	49%	53%	.77
Number of children in household			.81
1	32%	28%	
2	27%	36%	
≥ 3	41%	36%	
Primary caregiver’s educational attainment			.90
Not high-school graduate	63%	64%	
High-school graduate or at least some college	37%	36%	
Race/ethnicity of child			.94
Latino	66%	65%	
African-American	34%	35%	
Number of adults in household			.94
1	27%	31%	
2	46%	40%	
≥ 3	27%	29%	
Primary caregiver unemployed	58%	54%	.95
Type of insurance that primary caregiver has^c^			.95
Private	55%	40%	
Public	45%	54%	
Other	0%	6%	

^a^IPR_95_ denotes inner 95th percentile range

^b^Public health insurance in Mexico

^c^Among caregivers who have health insurance

**Table 5:** Multivariable analysis of factors associated with Medicaid/CHIP-eligible uninsured children remaining uninsured for the entire year

Characteristic	Adjusted Odds Ratio (95% Confidence Interval) for Remaining Uninsured for Entire Year
Child uninsured >6 months at baseline	3.8 (1.4, 10.3)
African-American/black race/ethnicity^a^	2.8 (1.1, 7.3)
Male child gender	2.4 (0.97, 5.8)
Primary caregiver employed	0.5 (0.2, 1.4)
Child born in US	0.2 (0.01, 3.7)
Combined annual family income below federal poverty threshold	0.4 (0.1, 1.4)

^a^Referent: Latino race/ethnicity

#### Costs

Uninsured children had significantly higher one-year mean costs (±SD) than children obtaining insurance for hospitalizations ($1131 ± 301 vs. $731 ± 122; *P* = .03) and wages and other costs of parental missed work days ($523 ± 111 vs. $126 ± 30; *P* = .04), and non-significant trends toward greater costs for ED visits and ICU stays (Table 6). Total one-year mean costs also were significantly higher for uninsured vs. insured children, at $5154.63 ± 1122 vs. $2268.88 ± 536 (*P* = .04). The resultant mean difference documents that the annual mean societal cost of caring for uninsured children is $2885.75 greater per year than for children obtaining insurance.

**Table 6:** Costs at one-year follow-up for uninsured children compared with children who obtained health insurance

Cost Item	Mean (±SD) Cost per Child		Difference	*P*
	Uninsured (*N* = 41)	Obtained Medicaid or CHIP Insurance (*N* = 196)		
ED visits	$607.14 (±213)	$476.92 (±400)	$130.22	.09
Hospitalizations	$1131.08 (±301)	$730.85 (±122)	$400.23	.03
ICU stays	$2893.63 (±557)	$934.86 (±356)	$1958.77	.10
Wages and other costs related to parental missed work days	$522.79 (±111)	$126.20 (±30)	$396.59	.04
Total costs	$5154.63 (±1122)	$2268.88 (±536)	$2885.75	.04

## Discussion

This prospective observational study demonstrates that providing health insurance to Medicaid/CHIP-eligible uninsured children results in significantly better health status; improved access to medical, preventive, and dental care; greater use of preventive services; a higher quality of well-child care; increased parental satisfaction; reduced out-of-pocket costs and family financial burden; and savings of approximately $2886 per year per child insured. The findings, thus, provide an answer to a longstanding crucial and fundamental question for researchers, clinicians, and policymakers: does providing health insurance to eligible uninsured children improve their health, healthcare, and outcomes, and save money? The study results provide evidence that insuring uninsured children achieves all of these benefits, while also reducing family financial burden and increasing parents’ satisfaction with their child’s healthcare.

The results address multiple research gaps by providing the first prospective cohort study of the impact of health insurance on eligible uninsured children, uniquely examining a wide variety of measures and providing the first comprehensive cost data. The findings complement prior work focused on CHIP (but not Medicaid or private insurance) predominantly from the 1990s and early 2000s, which employed pre/post telephone surveys (without control groups of uninsured children) and found that providing CHIP to previously uninsured, eligible children was associated with enhanced access to primary and specialty care; improved quality of care; enhanced parental and physician satisfaction with care; higher immunization rates; higher screening rates for anemia, lead, vision, and hearing; and reduced asthma ED visits and hospitalizations [9–11, 19, 25]. The combined evidence provided by prior research on CHIP and our current prospective observational results document the wide-ranging benefits of providing health insurance to Medicaid/CHIP-eligible uninsured children, including for health, healthcare, quality, parental and physician satisfaction, and cost savings.

The study findings indicate that insuring eligible uninsured children can have a powerful impact on population health. Health insurance proved to be a potent mechanism for obtaining a PCP: at baseline, 37% of uninsured children had PCPs, which substantially increased after 1 year to 82% for those obtaining health insurance, but dropped to 32% for those continuing to lack coverage. Data also document how coverage enhances preventive care: persistently uninsured children were 40 times more likely to have no source of preventive care, and insured children made almost twice as many preventive-care visits. Insurance coverage had a wide-ranging impact on sick care, including significantly increasing having a usual source of sick care, the same source of sick and preventive care, and access to 24-h telephone coverage for sick care, and boosting the number of sick visits to clinicians. Coverage also substantially influenced unmet healthcare needs, with insured children experiencing more than triple a reduction in overall unmet healthcare needs, over eight times fewer unmet needs for preventive care, and more than three times fewer unmet dental needs. The reduction in unmet dental needs is particularly noteworthy, given that the prevalence of dental caries in primary teeth is 51% in poor children and 44% in low-income children (family income 100–199% of the federal poverty threshold) 2–11 years old, and the prevalence of dental caries in permanent teeth is 66% in poor and 64% in low-income adolescents [26].

The patient experience—specifically quality and satisfaction—is one of the three pillars of the “triple aim” [27]. Obtaining insurance was associated with a significantly higher score for well-child-care quality, a greater likelihood of parents reporting that the doctor takes time to understand the child’s needs and respects the parent as the expert on the child, and a higher proportion of parents who would recommend the healthcare provider to friends. Although the reasons for these findings were not examined, one might hypothesize that these improved patient experiences may be related to a higher likelihood of insured children having key elements of medical homes, including a PCP, a usual source of preventive care, the same source of sick and preventive care, and the ability to get immediate care from PCPs.

Multivariable analyses revealed that Medicaid/CHIP-eligible minority uninsured children at greatest risk for remaining uninsured for at least 1 year are those who have been uninsured for at least 6 months at baseline and African-American children. These are the first published analyses, to our knowledge, to identify predictors of persistent uninsurance in children in a prospective cohort study. It can be hypothesized that children who have been uninsured for longer periods of time are at greatest risk for remaining uninsured over time, so these findings are not necessarily surprising, but the magnitude of quadruple the odds of remaining uninsured for those uninsured for >6 months is noteworthy. This also is the first study (to our knowledge) to document that African-American children have a significantly higher risk of remaining uninsured over time. The reasons for this higher risk are unclear, but may relate to qualitative research revealing that African-American parents of uninsured children report confusion and lack of knowledge about Medicaid/CHIP, cite many hassles with the Medicaid/CHIP application and renewal processes, and describe disrespect and related problems when seeking medical care for their children in the offices and clinics of Medicaid/CHIP healthcare providers [28]. Ultimately, our study findings suggest that special outreach and enrollment efforts targeting children uninsured for >6 months and African-American children might prove especially useful in reducing the large pool of uninsured children who are Medicaid/CHIP eligible.

Health insurance was found to substantially reduce the out-of-pocket costs of pediatric care, with mean out-of-pocket costs reduced by more than half per doctor visit, by a factor of seven for preventive-care visits, by a factor of five for sick visits, and by about half for ED visits. It therefore is not surprising that compared with parents of uninsured children, parents of children obtaining coverage were about six times less likely to report needing additional income to cover the child’s medical expenses, and four times less likely to report that the child’s health caused family financial problems. These findings underscore the major family financial burden of having an uninsured child. The results also complement prior work on Medicaid-eligible adults in Oregon which showed that Medicaid coverage was associated with reductions in any out-of-pocket spending, the amount of out-of-pocket spending, and financial strain from medical costs [29].

Certain study limitations and strengths should be noted. Study participants resided in urban communities in Dallas County, TX, so the findings may not necessarily generalize to non-urban settings or populations in other states. Participants were Latino or African-American, so the results may not necessarily apply to other racial/ethnic groups. A major study strength is that this is the first prospective observational study of the impact of health insurance on Medicaid/CHIP-eligible, uninsured children. This rigorous design provided a uniquely informative “natural history” approach to following a cohort of uninsured children, allowing for direct comparisons of children obtaining insurance versus those remaining uninsured over time. Such comparisons were not possible in prior studies, which either only followed newly insured children (but not the uninsured), or performed retrospective comparisons of children before and after obtaining coverage (without comparison to a cohort of uninsured children). Other study strengths include the frequent participant follow-up (monthly), independent verification of obtaining health insurance by HHSC, and the wide variety of outcomes assessed, including health status, access to care, unmet needs, use of services, quality, parental satisfaction, out-of-pocket costs, family financial burden, missed school and work, and societal costs.

Providing health insurance to Medicaid/CHIP-eligible uninsured children resulted in mean savings of approximately $2886 per child per year. One driver of these savings was the significantly lower costs of hospitalizations for insured children, at a mean difference of $400 lower per child per year. Data are not available to identify the reasons for lower costs for insured children, so additional study is needed of this issue; one could speculate that insured children’s greater access to PCPs, preventive care, and sick visits, coupled with lower unmet healthcare needs, may have resulted in shorter hospitalizations or admissions with lower severity of illness, hypotheses which may merit further investigation. The mean wage loss and other costs related to parental missed work days due to children’s illness also were significantly lower for children obtaining insurance, at a mean difference of $397 lower per child per year. This finding further underscores the employment and economic tolls on parents and families imposed by having an uninsured child.

The study findings have important federal policy implications. The results suggest that providing health insurance to all Medicaid/CHIP-eligible US children could save billions of dollars annually for our nation. Multiplying a cost savings of $2885.75 per child per year times the 3.0–3.5 million Medicaid/CHIP-eligible uninsured children in the US [5–8] yields potential annual savings of $8.7–$10.1 billion. But these savings cannot be realized until successful outreach and enrollment is achieved for these 3.0–3.5 million Medicaid/CHIP-eligible uninsured children. The Medicare Access and CHIP Reauthorization Act (MACRA) passed in early 2015 includes an additional $40 million to finance outreach and enrollment efforts to enroll more eligible children in Medicaid and CHIP [30]. MACRA also extends the Express Lane Eligibility option, which allows states to provide efficient Medicaid/CHIP enrollment and retention by using state income-tax data or findings on income and other Medicaid/CHIP eligibility factors from other programs deemed as Express Lane agencies, including the Temporary Assistance for Needy Families Program, Supplemental Nutrition Assistance Program, Head Start, Free and Reduced School Lunch Program, and Women, Infants and Children Program [30]. Randomized, controlled trials document that low-cost, highly effective interventions are available to insure uninsured children, including community-based case managers and parent mentors [14, 31]. Leveraging these available federal outreach and enrollment funds, Express Lane Eligibility options, and evidence-based interventions has the potential to improve the health, healthcare, care quality, and family finances for millions of eligible uninsured children, while potentially saving billions of dollars for our nation.

## Conclusions

This first-ever prospective cohort study documented that providing health insurance to Medicaid/CHIP-eligible uninsured children improves health, healthcare access and quality, and parental satisfaction; reduces unmet needs and out-of-pocket costs; and saves $2886/insured child/year. African-American children and those who have been uninsured for >6 months at baseline are at greatest risk for remaining uninsured for an entire year. Extrapolation of the savings realized by insuring uninsured children suggests that America potentially could save $8.7–$10.1 billion annually by providing health insurance to all Medicaid/CHIP-eligible uninsured children.

## Abbreviations

CHIP: Children’s Health Insurance Program
ED: Emergency department
ICU: Intensive care unit
LEP: Limited English proficiency
PCP: Primary-care provider
PedsQL: Pediatric Quality of Life Inventory Version 4.0 Generic Core Scales
RCT: Randomized, controlled trial
